# Magnetic nanorobots in oncology: From targeted drug delivery to smart theranostic systems

**DOI:** 10.1016/j.mtbio.2026.103709

**Published:** 2026-09-22

**Authors:** Xiaoyu Liu, Changying Li, Zhijie Xu, Yuanliang Yan, Wangrui Liu, Yong Li, Ming Gao

**Affiliations:** a Department of Gastrointestinal Surgery, Shandong Provincial Hospital Affiliated to Shandong First Medical University, Jinan, Shandong, 250021, China; b Medical Science and Technology Innovation Center, Shandong First Medical University & Shandong Academy of Medical Sciences, Jinan, Shandong, 250117, China; c School of Life Science, Shandong First Medical University & Shandong Academy of Medical Science, Tai'an, Shandong, 271016, China; d Integrative Center for Frontier Biotech and Pharmaceutical Sciences, Shandong First Medical University, Tai'an, Shandong, 271016, China; e State Key Laboratory of Environmental Chemistry and Ecotoxicology, Research Center for Eco-Environmental Sciences, Chinese Academy of Sciences, Beijing, 100085, China; f University of Chinese Academy of Sciences, Beijing, 100049, China; g Department of Pharmacy, National Clinical Research Center for Geriatric Diseases (Xiangya Hospital), The Hunan Institute of Pharmacy Practice and Clinical Research, Xiangya Hospital, Central South University, Changsha, Hunan, 410008, China; h Department of Pediatric Surgery, Hunan Children's Hospital, Changsha, Hunan, 410008, China; i Department of Thoracic Surgery, Renji Hospital, School of Medicine, Shanghai Jiao Tong University, Shanghai, 200127, China

**Keywords:** Nanomaterials, Magnetic nanorobots, Cancer treatment, Drug resistance, Tumor microenvironments

## Abstract

This review focuses on the application of magnetic nanorobots (MNRs) in tumor therapy. We first introduce the MNR-mediated drug delivery strategy, which loads anti-tumor agents and actively targets tumor sites. Leveraging the unique physicochemical properties of their constituent materials, MNRs enable controlled drug release in response to specific internal or external stimuli. Meanwhile, the near-infrared (NIR)-responsive components generate photothermal conversion for photothermal therapy (PTT) while released metal ions catalyze the Fenton reaction to produce reactive oxygen species (ROS), thereby inducing tumor cell death via chemodynamic therapy (CDT). Nevertheless, clinical translation of MNRs remains hindered by challenges related to biocompatibility, scalable manufacturing, real-time imaging and in vivo tracking. To address these issues, this review systematically summarizes recent representative studies on material design, new assessment system, and multimodal synergistic treatments. We also discuss emerging efforts integrating deep learning and artificial intelligence (AI) to optimize navigation and therapeutic efficacy. We aim to provide a comprehensive perspective on the path to clinical application by outlining current bottlenecks and potential solutions. This review is expected to offer new insights for further development of MNRs in precision cancer therapy.

## Highlights

• Non-fuel, non-invasive magnetic nanorobots for tumor treatment.
• MNRs integrate multiple treatment methods for multimodal cancer treatment.
• Controllable drug release and low cytotoxicity of MNRs enhance safety and efficacy.
• Reviewing clinical development and future prospects of MNRs in tumor therapy.

## Graphical abstract

**Figure:**
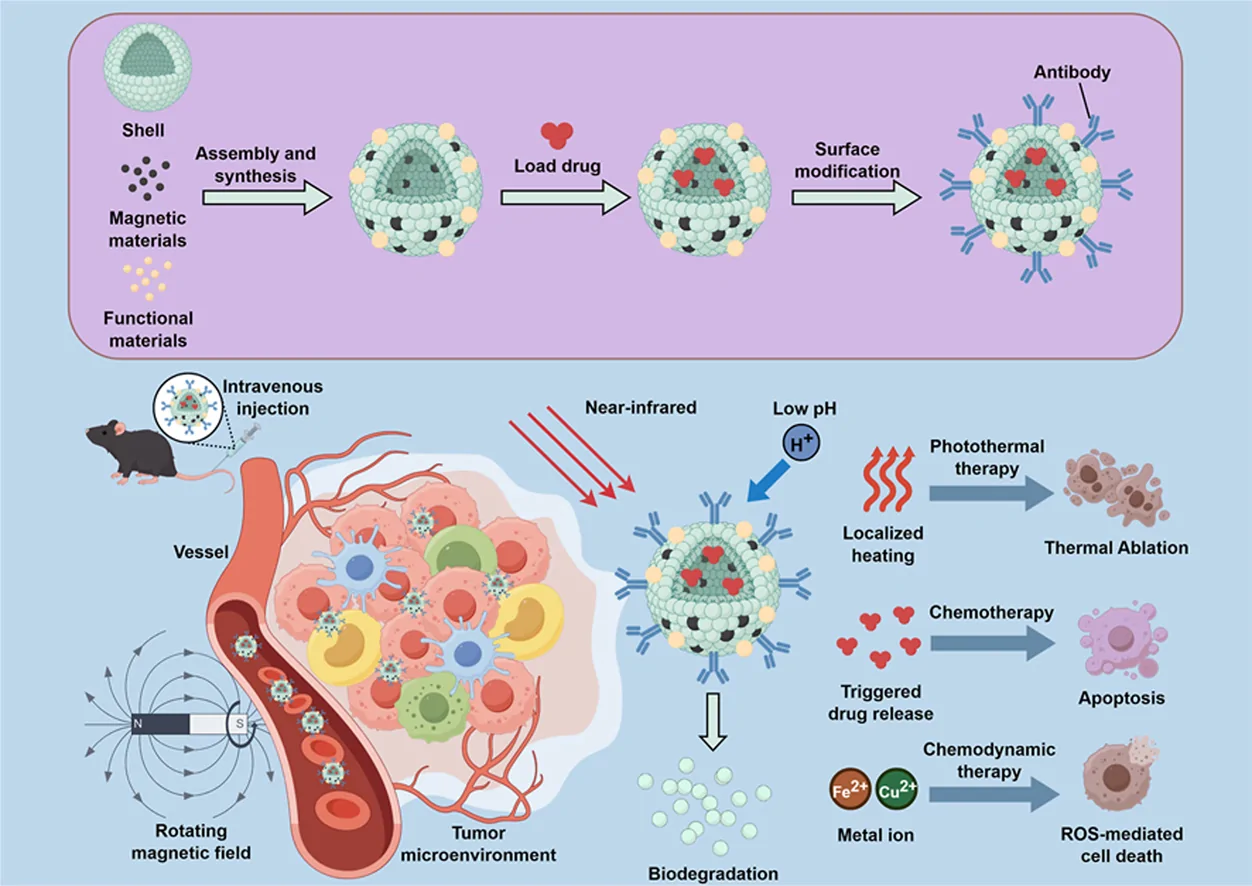

## 1 Introduce

Tumors are one of the leading causes of death globally, and their complexity and heterogeneity pose significant challenges to treatment. By 2050, the global annual incidence of cancer is projected to reach 35.3 million new cases, representing a 76.6% increase from the 20.0 million cases estimated in 2022. Likewise, cancer-related deaths are expected to rise to 18.5 million by 2050, corresponding to an 89.7% increase from the 9.7 million deaths recorded in 2022 [[1], [2], [3], [4], [5]]. In the field of tumor treatment, traditional surgery [6], radiotherapy [7,8], and chemotherapy [9,10] remain the mainstay, but emerging technologies such as immunotherapy [11,12], targeted therapy [13,14], and multimodal therapy [15] are rapidly evolving and gradually changing the treatment landscape. However, drug resistance [16,17], side effects [18], and the complexity of tumor microenvironment (TME) remain pressing issues to be addressed [19]. Currently, strategies such as multidisciplinary integration, precision medicine, and artificial intelligence aided diagnosis are continuously driving tumor treatment towards a more efficient and personalized direction.

Nanotechnology is a technology that operates and controls substances at the nanoscale, involving physics, chemistry, biology, materials science and other disciplines [[20], [21], [22]]. Its core is to develop new materials and equipment with unique properties by accurately manipulating atoms and molecules. The application of nanotechnology in the field of medicine is particularly striking, especially in tumor treatment, showing great potential. One of the most important applications of nanomaterials (NMs) is the targeting effect on tumors, which is divided into passive targeting and active targeting [[23], [24], [25]]. Due to the enhanced permeability of adjacent blood vessels caused by tumor tissue, NMs can target tumors through enhanced permeability and retention (EPR) effect. This targeting mode is passive targeting [26,27]. And the glycolytic effect of rapidly proliferating tumor cells will also cause the pH value near the tumor to decrease. Encapsulating small molecule drugs in pH sensitive NMs can release drugs near tumors, thereby improving the targeting of tumors [28]. However, the heterogeneity and dynamic nature of the tumor microenvironment (TME) pose significant challenges to the predictable performance of such responsive nanomedicines. To address this, emerging computational approaches such as physics-informed machine learning (PIML) have been developed to integrate biophysical principles with clinical data, enabling more accurate prediction of nanoparticle transport, drug release kinetics, and personalized therapeutic outcomes in complex TME [29]. Abraxane ® is a novel albumin binding paclitaxel 130 nm particle formulation, and paclitaxel is delivered in a suspension of albumin particles. It is approved for the treatment of recurrent breast cancer after combination chemotherapy, or breast cancer that recurs within 6 months after adjuvant chemotherapy. Its toxicity is also lower than that of standard paclitaxel [30]. Tumor cells express surface receptors that are different from normal cells [31]. Receptor mediated NMs targeting tumors utilizes these specific receptors, which may have a high margin of safety by reducing damage to normal tissues [[32], [33], [34]]. Therefore, significant progresses have been made in the application and development of nanotechnology in anti-tumor treatment, which provides new ideas and methods for drug delivery. Moreover, with the continuous innovation and improvement of nanotechnology, it is expected to bring more effective and safer treatment schemes for tumor patients.

The development of magnetic nanorobots (MNRs) can be traced back to the middle of the 20th century, but its real development and application are mainly concentrated in recent decades [35]. Early research mainly focused on the development of magnetic materials and magnetic field control technology [36]. After entering the 21st century, with the rapid development of nanotechnology, MNRs have made significant progress in targeted drug delivery, cell operation, minimally invasive surgery, biofilm destruction, environmental repair and other fields [37]. In recent years, researchers have developed a variety of MNRs with specific functions, such as MNRs for catalytic degradation of pollutants and antibacterial [38,39], and magnetic driven biological nanorobots for tumor targeted therapy [40]. The application of MNRs in tumor treatment can not only fully utilize magnetic fields to actively target tumors, but also release drugs as needed based on controllable factors or kill tumor cells at fixed sites. In addition to direct treatment, MNRs can also improve TME, and they have excellent biocompatibility and degradability (Fig. 1). Nanoparticles (NPs), as the most fundamental nanocarriers, rely entirely on the physical and chemical properties of the material itself or surface-modified targeting molecules for their function. Essentially, they are passive, static tools that rely on diffusion and cannot autonomously change their motion trajectory or apply external physical effects [41,42]. Other nanomedicine technologies, such as nano-scaffolds, biosensors, gene chips, and contrast agents, focus on building specific static interface environments or signal response platforms. Although they involve diagnostic and therapeutic functions, they lack controllable power systems and mechanical execution capabilities [43]. By contrast, driven by an external magnetic field, MNRs autonomously propel and rotate, thereby becoming programmable active units [44,45]. Beyond accurate navigation, they can perform dynamic physical tasks, such as cell perforation, tissue penetration, and mechanical force stimulation, thereby advancing nanomedicine from passive delivery/response to active intervention/manipulation [46].Furthermore, nanorobots can perceive and react to environmental stimuli, acting autonomously or under external control to perform predefined medical tasks [47,48]. These advantages make MNRs an excellent solution for addressing the complex challenges of cancer treatment. To ensure a systematic and comprehensive coverage of the literature, we performed a structured search in the PubMed, Web of Science, and Scopus databases using keywords including “magnetic micro/nanorobots”, “cancer”, “tumor” focusing on peer-reviewed articles published between 2015 and 2026. This review systematically summarizes the latest developments in MNRs for cancer treatment, including their broad prospects in clinical translation and collaboration with emerging technologies. We also focus on the application of MNRs in drug delivery, regulation of tumor progression, and therapeutic interventions, providing a new foothold and prospect for potential strategies in cancer managment.

**Fig. 1:**
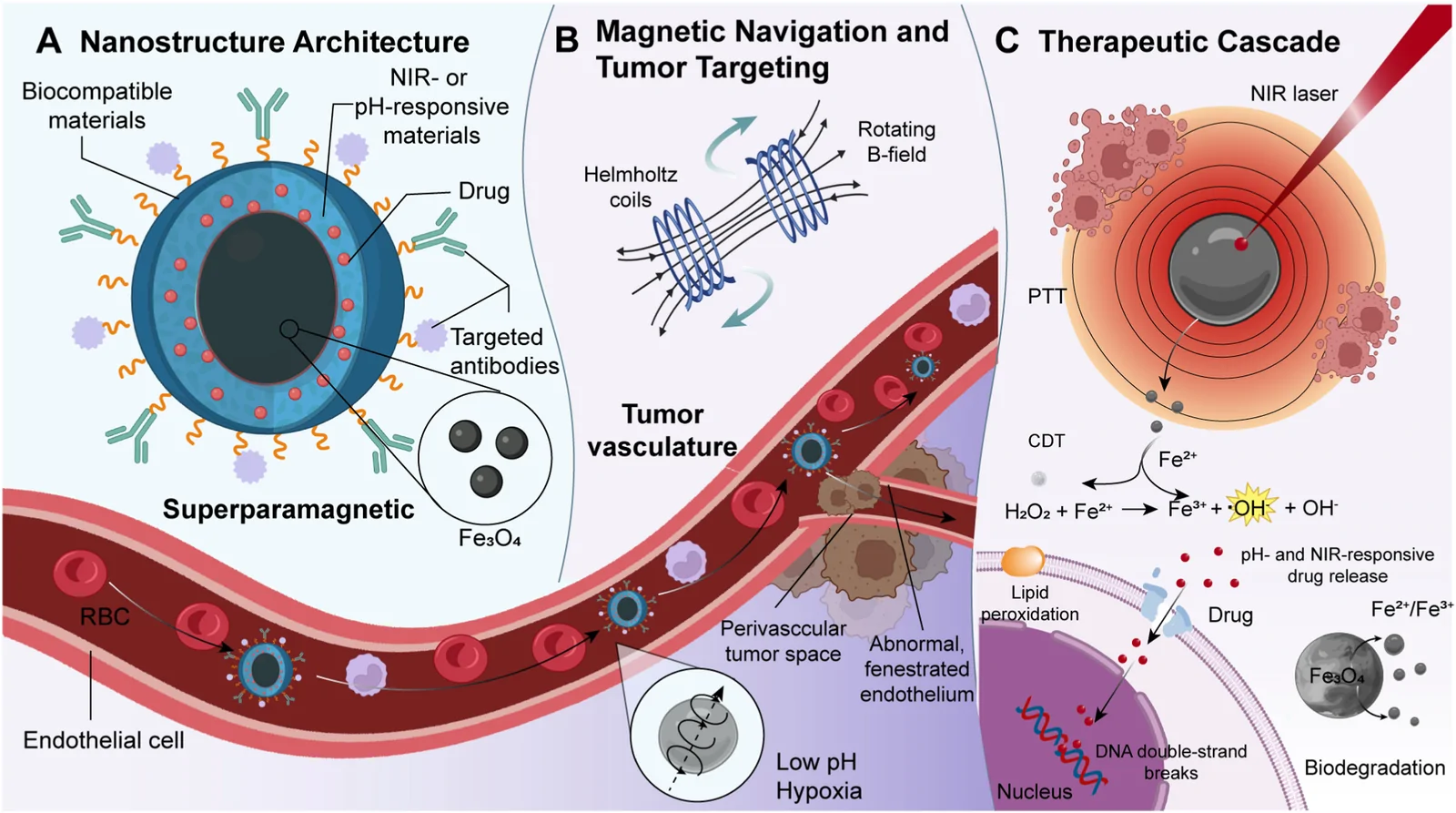
**The basic composition and function of MNRs.** (A) MNRs are generally composed of Fe_3_O_4_, which provides magnetism, biocompatible materials and materials responsive to NIR or pH. They can also carry drugs, and some have targeted antibodies on their surface. (B) MNRs can actively target tumor sites under the control of a rotating magnetic field. (C) MNRs can kill tumor cells through PTT and CDT, achieve controlled drug release at tumor sites, and ultimately achieve biodegradation.

## 2 Preparation principle, structural morphology and motion mode of MNRs

### 2.1 Synthesis and manufacturing of MNRs

The manufacturing of MNRs is the cornerstone that determines their morphological characteristics, material properties, and ultimately their movement behavior in biological environments [49]. Fabrication strategies are broadly classified into top-down and bottom-up approaches. Top-down manufacturing involves deconstructing large material blocks or structures and shaping or processing the material intensively to form the desired structure. This approach relies on physical or physicochemical subtractive processes, such as photolithography, three-dimensional (3D) printing/two-photon polymerization, focused ion beam (FIB) milling, and electron beam lithography (EBL), which excel in constructing complex 3D structures with high precision [50]. Conversely, bottom-up manufacturing builds materials or structures from the molecular or nanoscale level, typically starting from basic components such as molecules, NPs, or small building blocks and assembling them into larger systems. Representative bottom-up routes include self-assembly, chemical vapor deposition (CVD), DNA origami, and wet chemical synthesis [51]. For example, Shabbir et al. successfully achieved controllable self-assembly of nanoparticles by regulating the electrostatic interactions and non-covalent interactions such as π-π stacking between tripeptides and photosensitizer Ce-6. This strategy not only significantly improves the stability and biocompatibility of photosensitizers, but also enhances their photodynamic therapy (PDT) efficacy [52]. In terms of resolution, conventional photolithography is diffraction-limited (>100 nm), whereas high-resolution top-down techniques such as EBL can reach sub-10 nm resolution. However, these methods require expensive equipment and a clean-room environment, resulting in high costs. Meanwhile, top-down processing may leave residual photoresist or introduce ionic contamination, necessitating additional surface cleaning steps [53]. Bottom-up chemical synthesis, by contrast, offers atomic-level compositional control [54]. Wet chemical synthesis is characterized by relatively low cost and favorable scalability, while high-end routes such as CVD also require substantial capital investment [55,56]. Importantly, bottom-up approaches can directly introduce bioactive ligands (e.g., polyethylene glycol, PEG) during the synthesis stage, yielding clean surfaces with fewer defects and rendering them generally more amenable to in vivo applications [57].

The two strategies have distinct application scenarios in the fabrication of magnetic nanorobots. Top-down approaches provide precise geometric scaffolds with complex 3D architectures, making them more suitable for manufacturing magnetically controlled micro-helical swimmers. By contrast, bottom-up approaches endow chemical functionality, surface biofunctionalization, and nanoscale compositional control, rendering them more suitable for constructing magnetic nanoparticle swarms [58,59]. In biomedical and microrobotic applications, bottom-up methods are particularly valuable for creating MNRs for drug delivery and minimally invasive surgery. They are also applicable to dual-functional platforms integrating magnetic hyperthermia and drug release. For example, Fe_3_O_4_@SiO_2_@Drug core-shell structures synthesized via thermal decomposition, and drug release is triggered by the magnetocaloric effect [60]. A single fabrication route is often insufficient to meet the combined demands of structural complexity and multifunctionality. Hybrid fabrication is therefore emerging as a prevailing paradigm in this field. In a typical hybrid workflow, the structural chassis is constructed via top-down photolithography or self-rolling to ensure geometric accuracy and mechanical strength. Functional modules, including magnetic nanoparticles (MNPs), drug payloads and targeting ligands, are prepared via bottom-up chemical synthesis to retain material properties and biological activity [61,62]. The two components are subsequently integrated through microfluidic technology or surface chemistry strategies [63]. Ultimately, the synthesis method determines the structural precision and surface chemistry of MNRs, and also governs their magnetic responsiveness, drug-loading capacity, and degradation kinetics. In essence, the fabrication process lays the complete material foundation for subsequent locomotion, targeting, and cargo release.

### 2.2 Classification by geometric and structural

Unlike macroscale robots, MNRs exhibit unique motor behaviors owing to their distinct hydrodynamic characteristics [64]. Their design hinges on the interplay of geometric shape and material composition, which in turn determines their motion responses to external magnetic fields and forms the basis for motion control and optimization [65]. Among them, according to different shapes and assembly methods of robots, they can be divided into the following five categories (Fig. 2A): (i) Spherical MagRobots: Spherical MagRobots are spherical or spherical like magnetic microrobots, which are usually used in biomedical and environmental fields. The spherical or spherical like structure makes the robots have good motion performance in the fluid environment, and can move by rolling or sliding. Martin Pumera's team developed a multifunctional self-propelled PM/Pt Janus microrobot for biomedical applications. The Pt hemisphere of Janus microspheres provides a catalytic propulsion part, which can transport biomaterials ranging from 10 to 50 μm in complex cell culture media [66]. (ii) Helical MagRobots: Inspired by bacterial flagella, the helical structure enables micro/nano machines to convert rotational motion into translational helical motion by using a low-intensity magnetic field in low Reynolds number liquids. There are many ways to prepare spiral nanostructures. Li's team developed a helical magnetic nanomotor coated with human platelet plasma membranes to create a biomimetic biological interface. This platelet-derived coating endows the nanorobot with immune-evasion properties, thereby significantly prolonging its circulation time in the bloodstream. [67]. (iii) Flexible MagRobots: Flexible MagRobots are a kind of robots made of flexible materials with magnetic response ability. They can realize deformation, movement and function execution under the stimulation of external magnetic field. The flexible structure not only has the power advantage, but also can change the shape after receiving external stimuli. It has unique advantages for entering the complex or narrow areas that are difficult for the rigid structure nanorobots in medicine to reach. Rémi Dreyfus and his colleagues used biotinylated double stranded DNA as a “soft” hinge to connect red blood cells modified with superparamagnetic particles modified with streptavidin to prepare a new type of flexible artificial flagella through specific biotin streptavidin interaction [68]. (iv) Wire-like MagRobots: Wire-like MagRobots are a kind of magnetic microrobots with slender structure, which can realize movement and operation under the control of external magnetic field. It can walk flexibly in narrow and complex environments, showing a high degree of adaptability and controllability. Most rod-shaped magnetic robots are fabricated by template assisted electrochemical deposition (TAED). For example, a flexible Au/Ag/Ni nanowire, which has a gold “head” and a nickel “tail”, is connected by a partially dissolved and weakened silver bridge, which can realize fuel free magnetic drive propulsion and contribute to drug transport in the organism [69]. (v) Biohybrid MagRobots: Biohybrid MagRobots are a kind of microrobots that combine biological entities (such as microorganisms, cells or biomaterials) with magnetic components. They have achieved unique applications in biomedicine and other fields by integrating the natural characteristics of organisms and the functions of magnetic materials. For example, a core-shell structure Pd@Au (NPs) take Spirulina as its biological template, and the Fe_3_O_4_ NPs on its surface endow it with the characteristics of magnetic braking. These nanorobots can also be loaded with anticancer drugs to play the role of chemotherapy. It can not only carry out high-speed propulsion, but also has excellent photothermal treatment function [70].

**Fig. 2:**
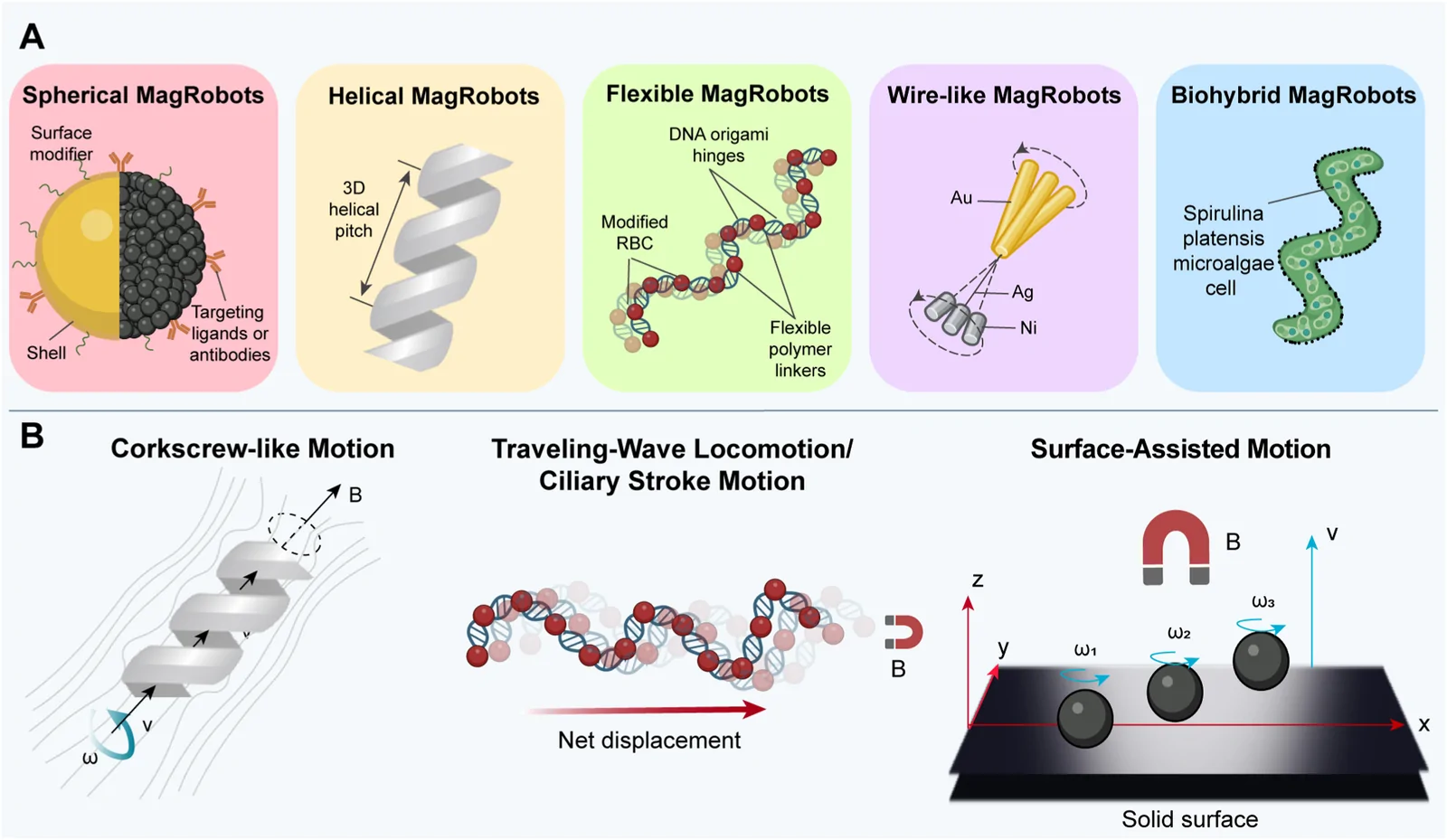
**Classification of MNRs.** (A) According to different shapes and assembly methods, MNRs can be classified into Spherical MagRobots, Helical MagRobots, Flexible MagRobots, Wire-like MagRobots and Biohybrid MagRobots. (B) According to the translation mechanism of magnetic micromachines, the propulsion methods of MNRs can be classified into Corkscrew-like Motion, Traveling-Wave Locomotion/Ciliary Stroke Motion, Surface-Assisted Motion.

### 2.3 Classification by locomotion kinematics

According to the translation mechanism of magnetic micromachines, it can be roughly divided into three types (Fig. 2B): (i) Corkscrew-like Motion: in nature, many microorganisms can coordinate their propulsion and orientation behavior with the motile appendages called flagella according to external stimuli. Eukaryotic cells (e.g., spermatozoa) can generate traveling wave motion by utilizing flexible beating flagella. In contrast, prokaryotic cells can perform helical movements by rotating their helical flagella. Inspired by the effective movement of bacterial flagella, artificial spiral micro/nano machines, known as artificial bacterial flagella (ABF) [71,72], are similar in shape to biological flagella and can generate thrust to swim under the control of a weak magnetic field. At present, a variety of such magnetic microrobots have been studied [73,74]. (ii) Traveling-Wave Locomotion/Ciliary Stroke Motion: Traveling-Wave Locomotion is a kind of movement mode that realizes the forward movement of organisms by continuously advancing waves in the medium. For example, some single celled organisms (e.g., Paramecium) or sperm cells move in this way. Dreyfus et al. constructed a magnetic colloidal chain acting as an artificial flagellum; under an oscillating field, it generated bending waves propagating from the free end toward the tether, propelling an attached red blood cell in the opposite direction (toward the free end) [68]. Ciliary Stroke Motion refers to the periodic actions experienced by cilia during movement, including Power Stroke and Recovery Stroke. For example, the cilia of epithelial cells in the respiratory tract help clear foreign bodies and mucus in the respiratory tract through rhythmic rowing. Kim et al. fabricated artificial ciliary microrobots that, under stepping magnetic fields, perform non-reciprocal power and recovery strokes, generating net thrust and enabling controlled swimming, precise steering, and targeted particle transport [75]. (iii) Surface-Assisted Motion: Surface-Assisted Motion is a way to achieve motion by utilizing surface properties or interacting with surfaces. This kind of motion mechanism usually involves an object or organism to promote its own movement with the help of surface friction, reaction force or other physical properties [76,77]. For instance, Park et al. developed magnetic surface microrollers that roll on solid surfaces under rotating magnetic fields; the nearby wall breaks flow symmetry to generate propulsion, and their 3D inclined locomotion is governed by a trade-off between hydrodynamic wall effects and gravity [78].

Magnetic drive has unique advantages in the driving methods of nanorobots. Firstly, magnetic drive can realize remote and spatiotemporal precise control without fuel, and has high reconfigurability and programmability. This driving mode allows magnetic fields to penetrate biological fluids effectively for remote actuation, unlike light- or chemical-based methods, even though the robots still face significant viscous resistance from the surrounding fluid [37]. Secondly, the magnetic driven nanorobots are reconfigurable and programmable. When a magnetic field or other external stimuli are applied, its shape, movement mode and other characteristics can be rearranged [79]. Finally, the magnetic drive also has the characteristics of recyclability. For Biosafety and biocompatibility, they can realize feasible and convenient magnetic assisted recovery and recovery process [80]. These advantages make magnetic drive have broad application prospects in the field of MNRs.

## 3 MNRs for drug delivery

### 3.1 Stimuli-responsive drug-releasing MNRs

One of the key challenges in advancing MNRs toward clinical application is whether drugs can be released in a controlled manner at the site of the lesion. In recent years, researchers have begun to incorporate the “stimulus-responsive concept” into magnetic nanocarriers: while retaining their magnetic targeting and magnetothermal advantages, these carriers achieve the benefits of “zero leakage, rapid response, and on-demand release” triggered by external light fields, endogenous pH gradients, or specific biochemical reactions [81] (Table 1).

**Table 1:** MNRs and BMMs that releases drugs in response to external or microenvironmental stimuli.

Release	Names	Core	Targeting	Therapy	Cancer	Refs
NIR response	CHMC	Hollow magnetic nanocarriers	Magnetic field-driven targeting	Chemo-photothermal therapy	Liver cancer	[82]
NIR response	Tri-bead/azo/DOX	NH_2_-Fe_3_O_4_ magnetic microparticles	Magnetic field-driven targeting	Chemo-photothermal therapy	Lung cancer	[83]
NIR response	Multifunctional nanorobot structure (MF-NRS)	Magnetic NPs-gold NPs-PDA composite systemstructure	Magnetic field-driven targeting	Chemo-photothermal therapy	Hepatocellular carcinoma (HCC)	[84]
pH response	CNT-DOX-Fe_3_O_4_	Fe_3_O_4_ NPs	Ligand-receptor mediated active and magnetic field-driven targeting	Chemotherapy	Colorectal carcinoma	[85]
pH response	Magnetically driven bionic drug-loaded nanorobots (MDNs)	Polyethylene glycol-modified iron oxide nanoparticles	Magnetic field-driven targeting	Chemo-immunotherapy	Breast cancer	[40]
Redox response	Magnetic cystine microrobots	Biodegradable cystine microparticles	Magnetic field-driven targeting	Chemotherapy	Prostate cancer	[86]
Ultrafast electroreductive mechanism	Bi/Ni/Pt	Trilayer Bi-based platform	Magnetic field-driven targeting	Chemotherapy	Breast cancer	[87]
pH response	BMMs	*Ch.*	Magnetic field-driven targeting	Chemotherapy	Cervical cancer	[88]
NIR and pH response	(Pd@Au)/Fe_3_O_4_ @*Sp.*-DOX	Pd@Au /Fe_3_O_4_ functionalized *Sp*. template	Magnetic field-driven targeting	Chemo-photothermal therapy	Renal cancer	[70]
Magnetothermal response	amNRs	Polyphosphate ester (PPE) soft matrix	Ligand-receptor mediated active and magnetic field-driven targeting	Chemotherapy	Breast cancer	[89]
Magneto-chemical dual response	Magnetic continuum robots (MCRs)	Silica NPs modified with urease and Fe_3_O_4_ NPs	Cross-scale hierarchical targeting	Chemotherapy	Glio-blastoma multiforme	[90]

MNRs can respond to near-infrared (NIR) light using photothermal conversion materials and convert it into thermal energy [91]. This thermal energy can induce the degradation or denaturation of drug carriers, thereby enabling precise and controlled drug release. To date, numerous reports have described the development of new drug delivery strategies. Taking advantage of the ability of chitosan to respond to NIR light [92], Chen et al. developed a chitosan-based hollow magnetic nanocarrier (CHMC) by surface modification of hollow mesoporous carbon (HMC) loaded with doxorubicin (DOX). Under NIR irradiation, the electrostatic interactions between chitosan and carbon materials are weakened, which enhances the mobility of drug molecules (Fig. 3A). Based on this property, CHMCs can be driven by a programmed magnetic field to achieve targeted delivery to cancer sites, NIR-responsive drug release, and synergistic antitumor effects [82]. The three-bead chiral microrobot is a controllable structure with excellent properties [93]. By incorporating a NIR-responsive molecule (an azo linker, 4,4′-azobis (4-cyanopentanoic acid)) into this structure, Song et al. designed new three-bead nanorobots. These MNRs are linked to the drug via strong covalent bonds, making them highly stable. Upon exposure to NIR light, the azo molecules decompose, releasing the anticancer drug and generating sustained localized heat at the target site [83]. Polydopamine (PDA) not only exhibits strong NIR light absorption and high photothermal conversion efficiency [94], but its surface-rich functional groups (catechol and amine) also provide sufficient drug-loading capacity [95]. Jin et al. prepared iron oxide magnetic nanoclusters (MNCs) using solvothermal method and deposited gold NPs on the surface of MNCs to produce MNC@Au. And PDA was subsequently coated to enhance the NIR light absorbance further and enable sufficient drug loading. These MNRs can release drugs in response to NIR light, demonstrating synergistic effects in both chemotherapy and photothermal therapy (PTT) [84].

**Fig. 3:**
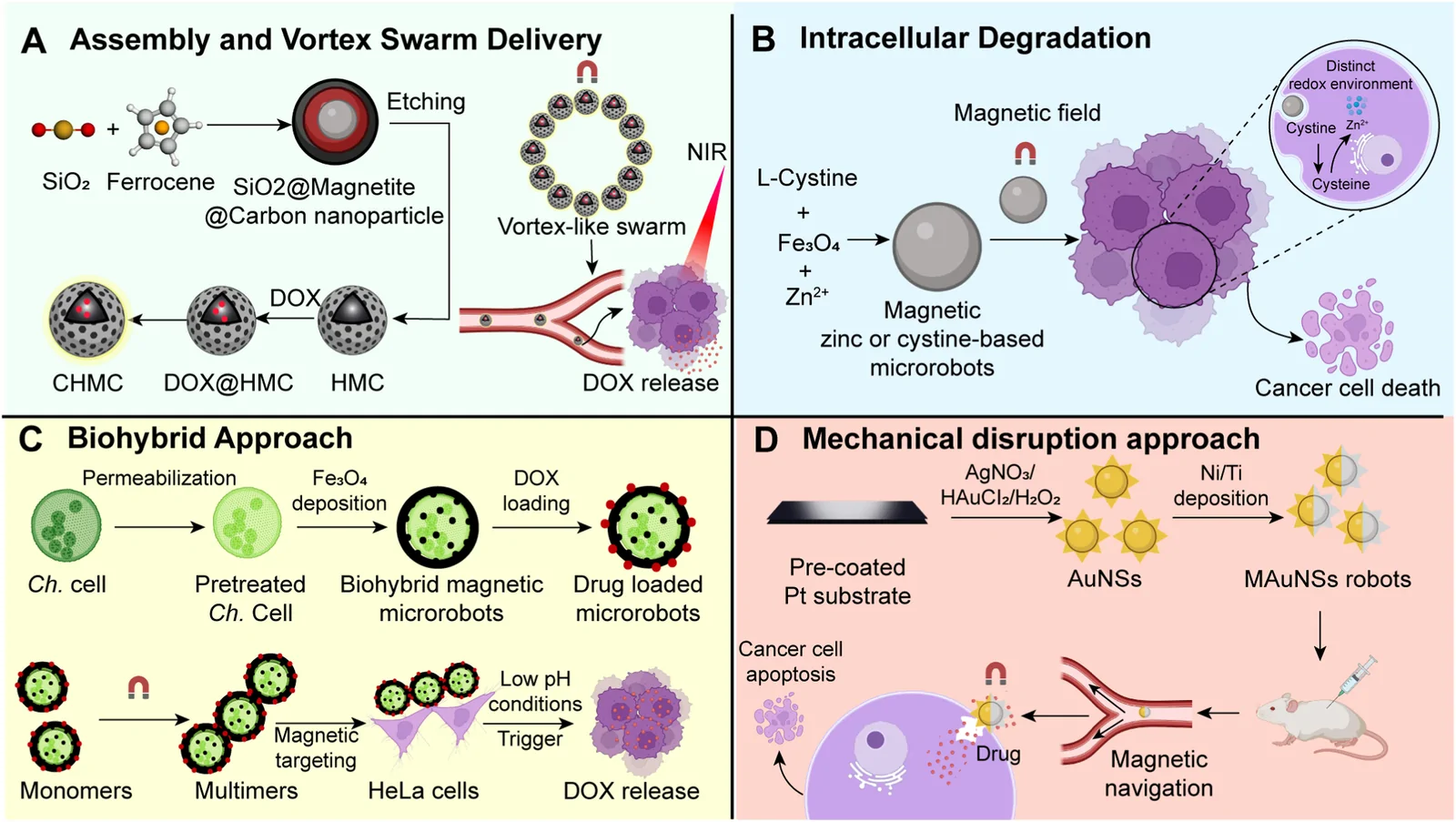
**MNRs with different administration methods. (A)** CHMC: chitosan-modified hollow mesoporous carbon loaded with DOX, featuring magnetic navigation and NIR-triggered drug release. **(B)** Cysteine microrobot: Fe_3_O_4_ nanoparticles self-assembled with cysteine via metal-ion mediation, magnetically steerable and redox-responsive. **(C)** BMMs: chitosan-based biohybrid microrobot chain fabricated by permeation stabilization, Fe_3_O_4_ deposition, and DOX loading, with pH-responsive drug release. **(D)** MNRs: Au nanorod-based nanorobots coated with Ni/Ti nanolayers for magnetic navigation and mechanically induced membrane permeabilization.

In the TME, the stability of hypoxia-inducible factor 1 (HIF1) increases due to reduced oxygen levels. Within this environment, glycolysis intensifies, leading to a lower extracellular pH compared to normal body tissues [96,97]. MNRs made from pH sensitive materials, upon reaching the target site, undergo chemical bond cleavage or structural protonation/deprotonation in response to a specific pH environment to release encapsulated drugs [98]. Predicting pH-responsive drug release kinetics is critical for delivery systems. Recent studies have employed machine learning to address this challenge. Rahdar et al. developed a neural differential equation (NDE) model that learns continuous-time release derivatives directly from data, identifying pH sensitivity as one of the primary determinants of release behavior [99]. Building on this, they constructed a physics-informed neural network (PINN) embedding Fickian diffusion and pH-dependent degradation equations, achieving high prediction accuracy (R^2^ = 0.96) for quercetin release across pH 5.4-7.4 [100]. Together, these frameworks offer mechanistically consistent tools for optimizing MNRs pH-responsive drug release. Andhari et al. chemically coordinated Fe_3_O_4_ NPs, coupled antibodies to carbon nanotubes (CNTs) through reactive spacer glutathione (GSH), and loaded anticancer drug DOX. This type of MNRs can exert their effects by breaking down H_2_O_2_ within the TME and pushing it deeper into the tumor [85]. In addition, the in situ oxygen generated by decomposing H_2_O_2_ may also help improve the hypoxic environment at the tumor site. Wang et al. developed a uniform ultrafine iron oxide NP based on polyethylene glycol modification, in which DOX is encapsulated by pH-responsive imine bonds. Researchers use a customized 3D magnetic manipulation platform (MMP) system to precisely control their movement patterns, which can adapt to changing and complex physiological environments, accurately target tumors, and deliver chemotherapy drugs. This type of MNRs can activate the immune response at the tumor site, significantly prolonging the survival time of mice [40].

In addition to releasing drugs through photothermal effects and pH response, some MNRs can also achieve drug release at the tumor site through biological or chemical reactions [101]. Previous studies have shown that the metabolic differences of zinc in prostate cancer are an important diagnostic biomarker and potential therapeutic target [102]. Ussia et al. proposed a Zn^2+^ mediated cysteine based self-degrading microrobot, which is made by self-assembly of superparamagnetic Fe_3_O_4_ nanoparticles encapsulated in amino acid cysteine and can be precisely manipulated through a rotating magnetic field. After reaching tumor cells, this type of microrobot is absorbed by the cells, and the intracellular enzymatic reduction environment leads to the degradation of cysteine, thereby releasing the Zn^2+^ that make up the microrobots (Fig. 3B) [86]. In addition to zinc, bismuth (Bi) metal has become an ideal metal for manufacturing micro robots due to its chemical stability in water, good biocompatibility, and high surface area [103]. The bismuth based tubular micro robots can carry DOX and perform self-propulsion. After reaching the tumor site, this MNRs can release drugs through an electrochemical reduction mechanism, injecting electrons into the MNRs, which allows for ultrafast drug release when a low cathodic potential is applied. The effectiveness of this method has been demonstrated in vitro experiments. Compared to pH-responsive release methods, this approach can release drugs faster [87].

The aforementioned studies have demonstrated that MNRs possess the potential for anticancer drug loading and on-demand release. Notably, a machine learning-driven optimization study has identified rational formulation parameters for nanocarrier design, providing a data-driven basis for translating in vitro cytotoxicity into clinically viable MNR formulations [104]. However, limitations also objectively exist. MNRs with photothermal response have insufficient deep penetration into tumor tissue, and the high temperature of photothermal conversion may also damage normal tissue [105]; The weakly acidic environment of TME may affect the sensitivity of pH-responsive MNRs and lead to off target drug release [106]; MNRs that respond to biochemical reactions also face the issue of heterogeneity in the tumor tissue environment. These issues still need to be properly resolved in the future.

### 3.2 Biohybrid magnetic microrobots

Biohybrid magnetic microrobots (BMMs) are a type of micro robotic system that integrates natural biomaterials (such as microalgae, bacteria, mammalian cells, etc.) with magnetic NMs (such as Fe_3_O_4_ NPs, superparamagnetic iron oxide NPs (SPION), etc.) [107]. This type of robot combines the high biocompatibility, low immunogenicity, and special biological functions of biological components, as well as the controllable navigation characteristics of magnetic materials (Table 1).

Natural biological templates have good biocompatibility and fine structure, providing new ideas for the preparation of magnetic micro/nanorobots. Chlorella (*Ch.*) is a single celled microalgae that is smaller in size than human red blood cells. Compared to other larger and more difficult to degrade biological templates, it has good biocompatibility and biodegradability [81,108]. Gong et al. sequentially subjected *Ch.* to permeation stabilization, Fe_3_O_4_ deposition, and DOX loading to manufacture BMMs (Fig. 3C). Using dynamic magnetic self-assembly technology, these microrobots units can be driven to separate and construct a biohybrid microrobot chain. This type of BMMs exhibits excellent propulsion performance and maneuverability at low Reynolds numbers. After reaching the tumor site, BMMs can release DOX in response to pH, achieving precise response release [88]. In addition to *Ch.*, Spirulina (*Sp.*) has also been applied in the production of BMMs in the same way. Spirulina is a microorganism with a natural and complete three-dimensional spiral structure, which also has good biocompatibility and can serve as an ideal biological template for drug loading and targeted delivery [109]. Wang et al. proposed a strategy for constructing magnetic microrobots using the biomaterial *Sp.* as a scaffold. The Fe_3_O_4_ NPs on the surface of the robots enable it to be controlled by a magnetic field. It simultaneously loads DOX and the one with photothermal conversion capability Pd@Au core−shell NPs, to achieve synergistic therapeutic effects. These advantages make it a very promising and effective platform [70].

In addition to the direct use of biological templates, there is also bionic magnetic nanorobot, which takes inspiration from naturally occurring biological structures and imitates them. Many nanoplatforms encounter the problem of being unable to efficiently pass through biological barriers when delivering drugs. And according to the Cannikin law, the overall drug transport efficiency of magnetic nanoplatforms is always limited, ultimately leading to poor therapeutic effects of drugs on tumor tissues [110]. Inspired by the unique deformation ability of amoeboids to actively bypass obstacles, Zhu et al. developed a magnetic driven amoeboid like nanorobot amNR to achieve full process active drug transport [89]. The core of the robot is composed of polyphosphoester (PPE) with low glass transition temperatures (T_g_s), which has unique deformability and can actively infiltrate from blood vessels into deep tumor tissues through magnetic driven deformation effects. Finally, the nanorobot generates heat under an alternating magnetic field (AMF), triggering rapid drug release. To achieve higher coverage of active transportation during drug delivery, Wu et al. developed a two-stage active targeting marsupial robot integrated system for the treatment of glioblastoma (GBM). This system includes a mother robot and child robot. These dual responsive nanorobots were prepared using dendritic mesoporous silica NPs (DMSNs) modified with urease and Fe_3_O_4_ NPs [90]. After macro targeting, the nanorobot is released from the micro channel of the mother robot after bypassing the blood-brain barrier (BBB). Performing more precise secondary targeting at a smaller microscale to improve the targeting rate and therapeutic efficacy of drug delivery. Meanwhile, its strong DOX loading capacity and rapid pH responsive drug release also make it an excellent drug carrier. These MNRs provide a promising treatment option for minimally invasive local treatment of GBM. Although there are still issues to be addressed, such as manufacturing efficiency and homogenization, real-time tracking, and imaging, the excellent biocompatibility and degradability of magnetic BMMs will have wide applications in medical diagnosis, treatment, and drug delivery in the future.

### 3.3 Other ways of drug delivery by MNRs

In addition to using materials that respond to external physiological environments or stimuli, and using biological templates or biomimetic structures to deliver drugs, researchers are also exploring new ways of drug delivery. In transmembrane drug delivery, small molecule drugs usually cross the cell membrane by passive transport. However, this process will be subject to many limitations [111]. Therefore, there is an urgent need to develop innovative transmembrane delivery mechanisms to improve clinical drug delivery efficacy. Therefore, in addition to improving the physical and chemical properties of drugs or modifying the surface of drug molecules, Liu et al. creatively proposed a drug delivery strategy that directly affects the cell membrane. They developed MNRs whose main body is composed of nanoscale Au nanorods coated with Ni and Ti nanolayers. The Ti nanolayer thus acts as a protective barrier against Ni release and concurrently ensures biocompatibility [112]. Under the control of an external magnetic field, these MNRs can accurately navigate to target cancer cells and apply mechanical agitation on the cell membrane to promote intracellular drug delivery [113]. Due to their sharp nanorod structure, the magnetic driven rotational behavior of these MNRs can apply mechanical agitation to the living cell membrane, thereby increasing the permeability of the cell membrane and promoting the transport of transmembrane cargo (Fig. 3D). This work opens up a new avenue for using MNRs systems to overcome the cell membrane barrier of drug delivery within cells, and is expected to promote the further application of magnetic nanorobot technology in the field of precision medicine.

Abnormal vascular structure and impaired blood flow in TME are important reasons for the poor delivery of anti-tumor drugs [114]. Minoxidil (MXD) is an AT-P sensitive potassium channel opener and vasodilator that can promote vasodilation, reduce hypoxia, and facilitate drug delivery, providing the potential to regulate TME [115,116]. Encouraged by this, Mohammadzadeh et al. proposed a drug delivery strategy using Minoxidil Nanoliposomes (Lip-MXD) to induce tumor angiogenesis and enhance the delivery of PEGylated liposomal DOX (PLD) [117]. Lip-MXD can significantly enhance PLD permeability in TME through its vasodilatory effect. Single administration of PLD after Lip-MXD pretreatment showed significant anti-tumor activity and improved survival rate. This strategy provides new ideas for the delivery of tumor drugs. In addition to strategies that utilize physiological changes caused by drugs to enhance drug delivery, there are also strategies that utilize physiological changes caused by other treatment methods to promote drug delivery. Vascular targeted photodynamic (VTP) therapy is a method of eliminating cancer cells by targeting the vascular septa of tumors [118]. Nogueira et al. found that inflammation induced by VTP increases the expression of P-selectin on tumor blood vessels. They loaded anti-cancer drugs onto P-selectin NPs and used them in conjunction with VTP [119]. This combination therapy significantly enhances local tumor control and increases recurrence free survival compared to monotherapy. Simultaneously preserving healthy tissues. This VTP guided nanoparticle delivery method provides a promising strategy for future clinical trials.

## 4 MNRs in tumor microenvironment

The occurrence and development of tumors is a dynamic process involving multiple steps and factors [120]. The entire process begins with a key gene mutation in a single cell, and ultimately transformation into a malignant phenotype. This process provides a modifiable window period for clinical intervention, and many MNRs have been proposed to address this process (Table 2). In the stage of tumor development, continuous proliferation, apoptosis escape, and metabolic reprogramming occur [121], so intervening in its metabolism may be a good choice. Mitochondria, an organelle closely related to cellular metabolism and endogenous apoptosis, are a potential option for cancer treatment [122]. Triphenylphosphine (TPP) is a classic mitochondrial-targeting moiety that accumulates in mitochondria driven by the negative membrane potential [123]. Fucoidan (FU) specifically binds to P-selectin, which is overexpressed on tumor vascular endothelial cells, thereby enabling active targeting of the tumor vasculature [124,125]. By integrating both TPP and FU into a single nanoplatform, the system achieves dual targeting. FU@TPP can deliver pterostilbene (PTE) and Mn^2+^. The in vitro experimental results showed that these MNRs can induce mitochondrial related apoptosis and activation of the cyclic guanosine monophosphate-adenosine monophosphate synthase-stimulator of interferon genes (cGAS-STING) pathway in 4T-1 cells. In vivo experiments showed that tumor growth was inhibited and tumor lung metastasis was reduced [126].

**Table 2:** MNRs that improve TME or regulate immunity.

Names	Core	Functions	Refs
FU@TPP/PTE Mn^2+^	FU@TPP/PTE Mn^2+^ NPs	Dual targeted mitochondrial dysfunction induction and cGAS-STING activation	[126]
Fe_3_O_4_@nSiO_2_-mSiO_2_-CAT/LOX/TP/ICG-AS1411	Superparamagnetic Fe_3_O_4_ NPs	Enzymatic cascade reaction for hypoxia-alleviating targeted drug delivery	[127]
MRs	Nano-smooth drug-loaded system with M1 macrophages	Inducing macrophage M1 polarization and drug delivery	[128]
DOX@MPN	M1 macrophage membrane-coated magnetic photothermal nanocomplexes	Drug delivery, PTT and macrophage-based immune regulation	[129]
MCRs	Macrophage loaded with DOX/ICG-functionalized magnetic nanoparticle	Macrophage-based natural tumor targeting, drug delivery and PTT	[130]
Mag-C	Magnetic chitosan microscaffold with Fe_3_O_4_ nanoparticles embedded in chitosan matrix.	Vessel embolic effect, real time imaging and transport of macrophages	[131]
OVA-CaCO_3_-SPIO (OCS)	OVA-CaCO_3_-SPIO composite structure	Neutralizing TME acidity, promoting lysosomal escape and cross antigen presentation	[132]

TME is a complex multicellular environment in which tumors are located, mainly composed of immune cells [133,134], stromal cells [135], extracellular matrix [136], secreted small molecules, as well as blood and lymphatic vessel networks, and is highly heterogeneous and dynamic [137]. The proliferation, immune escape, and distant invasion of tumors are closely related to TME, and in turn affect TME [138]. Scientists have confirmed through over 60 years of clinical and experimental evidence that 90% of solid tumors suffer from hypoxia, which is considered one of the hallmarks of cancer [139]. Tumor cells have a higher proliferation rate and metabolic activity, and their oxygen demand often exceeds their oxygen supply [140,141]. Tumor cells adapt to hypoxia by altering their signaling pathways [142]. Hypoxia can promote the malignant behavior of cancer cells. Hypoxia can stimulate an increase in glycolysis in TME, leading to an increase in lactate and H^+^ levels in TME, resulting in a lower pH in tumor tissue compared to normal tissue [143,144]. This heterogeneous environment of hypoxia and low pH is also a significant obstacle to tumor drug delivery. Interestingly, this same hypoxic TME also offers opportunities for MNRs design: by integrating oxygen-consuming or oxygen-generating enzymes, MNRs can achieve self-propulsion while simultaneously alleviating local hypoxia. Liu et al. constructed a cascade enzymatic system by co-loading lactate oxidase (LOX) and catalase (CAT). In this system, LOX consumes lactate to alleviate the acidic TME, while the subsequent decomposition of produced H_2_O_2_ by CAT generates oxygen bubbles to propel the nanorobots for deep tissue penetration, collectively enhancing therapeutic efficacy. In addition, the MNRs can also load Triptolide (TP) and modify AS1411 aptamers to achieve active targeted drug delivery [127].

In addition to improving the physiological environment of TME, immune cells have received attention due to their importance in regulating the TME of tumor progression. Macrophages, as one of the main components of immune cells in TME, have also received attention. According to their function and phenotype, macrophages can be divided into two types: macrophages M1 and M2. However, it is important to note that macrophage polarization in vivo is highly dynamic and context-dependent, with cells often exhibiting mixed phenotypes that span the M1-M2 spectrum rather than existing strictly as discrete subsets [145]. According to reports, the polarization of macrophages is influenced by the properties of the attachment surface [146]. Therefore, leveraging the surface properties of MNRs, such as topography, charge, or chemical composition, may provide a means to modulate macrophage polarization toward the anti-tumor M1 phenotype, thereby contributing to a more favorable therapeutic TME. Studies have shown that surface topography can influence macrophage polarization; for instance, certain rough surfaces may promote the pro-tumor M2 phenotype, whereas relatively smooth surfaces tend to favor the anti-tumor M1 phenotype [147]. Based on this characteristic, Song et al. designed a nanorobot that provides a flat, nanoscale smooth surface, which can induce macrophages to polarize towards anti-tumor phenotype [128]. Afterwards, they also developed a magnetic photothermal nanocomposite (MPN) wrapped in M1 macrophage membrane. MPNs can target tumors by leveraging the innate tumor-homing and immune properties of macrophages [129]. Both of these platforms can be loaded with DOX for targeted delivery. The latter can also generate high levels of reactive oxygen species (ROS) under NIR irradiation. In addition, adhering magnetic materials to the surface of macrophages or entering the interior of cells allows macrophages to be controlled by magnetic fields. Using macrophages with better biocompatibility to transport drugs through this system is also a good method. Dai et al. proposed a magnetized cell-based robot (MCR) based on macrophage drug carriers [130]. Living macrophages are converted into MCRs through endocytosis of specially-designed MNPs loaded with DOX and indocyanine green. After MCRs target the tumor site, they can not only generate heat for PTT under NIR irradiation, but also release the loaded DOX. Notably, even without NIR irradiation, the MCRs themselves exhibited an M1-like phenotype or could induce the polarization of tumor-associated macrophages (TAMs) toward the antitumor M1 phenotype. In addition to the design that allows macrophages to engulf magnetic substances and drugs as carriers, there is also the idea of using MNRs to transport macrophages. Go et al. fabricated the magnetic chitosan microscaffolds (Mag-C) with optimized shape and pore properties to specific target diseases. Mag-C consists of a chitosan microscaffold (CMS) and magnetic particles (MPs) attached to its surface [131]. Mag-C utilizes its highly biocompatible chitosan material and adjustable porous structure to enhance macrophage adhesion, and acts as a programmable magnetic microscaffold to achieve magnetic field guided delivery of macrophages.

As is well known, the effective processing of antigens by antigen-presenting cells (APCs) is the first step in initiating an immune response [148]. However, in the acidic TME, this process cannot fully perform its intended function. On the one hand, due to the inactivation of APCs in the acidic TME, the presentation of endogenous tumor antigens is impaired, failing to adequately activate the immune system [149,150]. On the other hand, exogenous antigens in vaccines are usually internalized into lysosomes and presented to CD4^+^ T lymphocytes by MHC II molecules, predominantly triggering humoral responses unless they are diverted into the cross-presentation pathway to activate CD8^+^ T-cell-mediated cellular immunity. [151]. To address this issue, Ye and colleagues designed a magnetically driven OVA-CaCO_3_-SPIO robot (OCS robot). This robot can be controllably guided to the tumor site in a magnetic field, neutralizing the acidity of the TME. Meanwhile, it can also restore the activity of dendritic cells (DCs), promote lysosomal escape and cross antigen presentation, thereby triggering cellular immune responses [132]. Beyond macrophages and DCs, other immunosuppressive populations, such as myeloid-derived suppressor cells (MDSCs) and regulatory T cells (Tregs), also play critical roles in the TME [152,153]. Although their interaction with MNRs remains largely unexplored, they represent an important direction for future investigation. In summary, MNRs are providing new potential solutions for tumor suppression and prognosis improvement during this important stage of tumor occurrence and development.

## 5 MNRs for tumor treatment

When the magnetic nanorobots accurately locate to the TME by virtue of magnetic navigation, how to convert local energy or drugs into high-efficiency, low toxicity and sustainable therapeutic effects has become the core proposition that determines its clinical fate. PTT and chemodynamic therapy (CDT) are the main methods of tumor treatment by MNRs at present (Table 3).

**Table 3:** MNRs for tumor treatment.

Names	Core	Targeting	Therapy	Cancer	Refs
Fe_3_O_4_-Azo-DOX (FAD)	DOX-grafted magnetic nanorobot collectives	Magnetic field-driven targeting	Chemo-photothermal therapy	Peritoneal metastaticr	[145]
BMPNs	PPy@F nanocomplex coated with platelet/M1 macrophage hybrid membrane	Biomimetic membrane and magnetic field-driven targeting	CDT/chemotherapy /PTT/immune checkpoint blockade combination	Glioblastoma multiforme	[147]
*Ch.*@Fe_3_O_4_@ PDA@ZIF-8 (CFPZ)	Chlorella-Fe_3_O_4_-PDA-ZIF-8-DOX composite	Magnetic field-driven targeting	Chemo-photothermal therapy	Cervical and liver cancer	[148]
FeO@mSiO2/Au-CAT JNCRs	Janus FeO@mSiO_2_/Au NPs	Self-propelled chemotactic targeting	Chemo-photothermal therapy	Breast cancer	[154]
MNRs@GOx/Cu/HSA	Linearly arranged magnetic Fe_3_O_4_ NPs connected by metal-polyphenol complexes	Magnetic field-driven targeting	Chemodynamic therapy	Breast cancer	[155]
Cu@MPS-GOD	Cu^2+^-doped magnetic mesoporous silica nanoparticles loaded with GOD	Magnetic field-driven targeting	Chemodynamic-immunotherapy	Breast cancer	[156]
superparamagnetic PolyHb-Tyr-nano	Nanoencapsulated Polyhemoglobin-Tyrosinase (PolyHb-Tyr) complex	Magnetic field-driven targeting	Amino acid depletion and oxidative stress therapy	Multiple cancer	[157]

### 5.1 Photothermal therapy

PTT is a novel tumor local treatment strategy that utilizes photothermal agents to convert NIR light energy into thermal energy and induce tumor cell death through local thermal ablation. Compared with traditional surgery, radiotherapy, and chemotherapy, PTT has outstanding characteristics such as minimally invasive nature, high selectivity, and spatiotemporal controllability [158]. In addition, PTT can induce immunogenic cell death (ICD) in tumor cells, characterized by the release of damage-associated molecular patterns (DAMPs), thereby stimulating anti-tumor immune responses. However, PTT still faces problems such as limited tissue penetration depth of NIR light and difficulty in reaching deep tumor areas with photothermal agents, which limits its complete tumor ablation efficacy in complex TME. MNRs provide a new technological path to overcome the aforementioned bottlenecks of PTT. The core design concept is to integrate magnetic navigation, photothermal therapeutic capability, and imaging functionality into a single nanoplatform, enabling precise targeted delivery and synergistic therapy [159].

The combination of hyperthermic intraperitoneal chemotherapy (HIPEC) and cytoreductive surgery (CRS) is still the main method for treating peritoneal metastatic cancer [160,161]. Wang et al. reported a laser integrated magnetic actuation system. This system comprises silica-coated Fe_3_O_4_ nanoparticles (Fe_3_O_4_@SiO_2_ NPs), which can target tumor sites to enhance local photothermal effects and achieve rapid hyperthermia [162]. At the same time, these NPs are also linked to DOX through aliphatic azo groups that break down at temperatures exceeding 45 °C, allowing for controlled local drug release and reducing off-target tissue damage at elevated temperatures. Ultimately, synergistic treatment of thermochemotherapy is achieved at the tumor site. The presence of BBB is an important factor hindering the thermal therapy of GBM [163]. In order to enhance BBB penetration and target GBM, Song et al. introduced a hybrid membrane derived from platelets and M1 macrophages into the structure of the MNRs. This biomimetic magnetic nanorobot is encapsulated with polypyrrole/Fe_3_O_4_ nanocomplex (PPy@F) for PTT and promote the Fenton reaction of Fe_3_O_4_ to generate CDT. In addition, MNRs also carry temozolomide and PD-L1 antibody (SNTSESF) as chemotherapy drugs and immune checkpoint inhibitors, respectively. This multifaceted therapeutic regimen, PTT, chemotherapy, CDT, and immune checkpoint blockade (ICB), offers a promising strategy for the treatment of GBM (Fig. 4A). [164]. This type of nanorobot can improve tumor targeting, enhance tumor immune efficacy, and actively target in situ gliomas in mice. In addition, metal-organic frameworks (MOFs) with large specific surface area and good scalability have been explored as alternative materials for biomedical applications [154]. On the one hand, zeolitic imidazolate framework-8 (ZIF-8) has low cytotoxicity and pH responsive degradability, which makes it a potential drug carrier in acidic biological environments [155,156]. But its further application faces problems of poor dispersion and lack of targeting. On the other hand, the abundant catechol groups in PDA can promote heterogeneous nucleation and growth of MOFs on PDA surface [165]. Using the characteristics of PDA, Gu et al. proposed a novel strategy for mass production of MOF-loaded biotemplated magnetic microrobots based on *Ch.* [166]. They deposited superparamagnetic Fe_3_O_4_ NPs inside microalgae cells (*Ch.*@Fe_3_O_4_) Then, PDA was modified and ZIF-8 NPs were further grown in situ to prepare *Ch.*@ Fe_3_O_4_@PDA@ZIF-8 (CFPZ) microrobots, which enabled precise propulsion under a rotating magnetic field and rapid PTT upon NIR irradiation (Fig. 4B). The resulting CFPZ microrobots exhibits strong photothermal conversion capabilities and can also carry DOX and enable on-demand release of DOX.

**Fig. 4:**
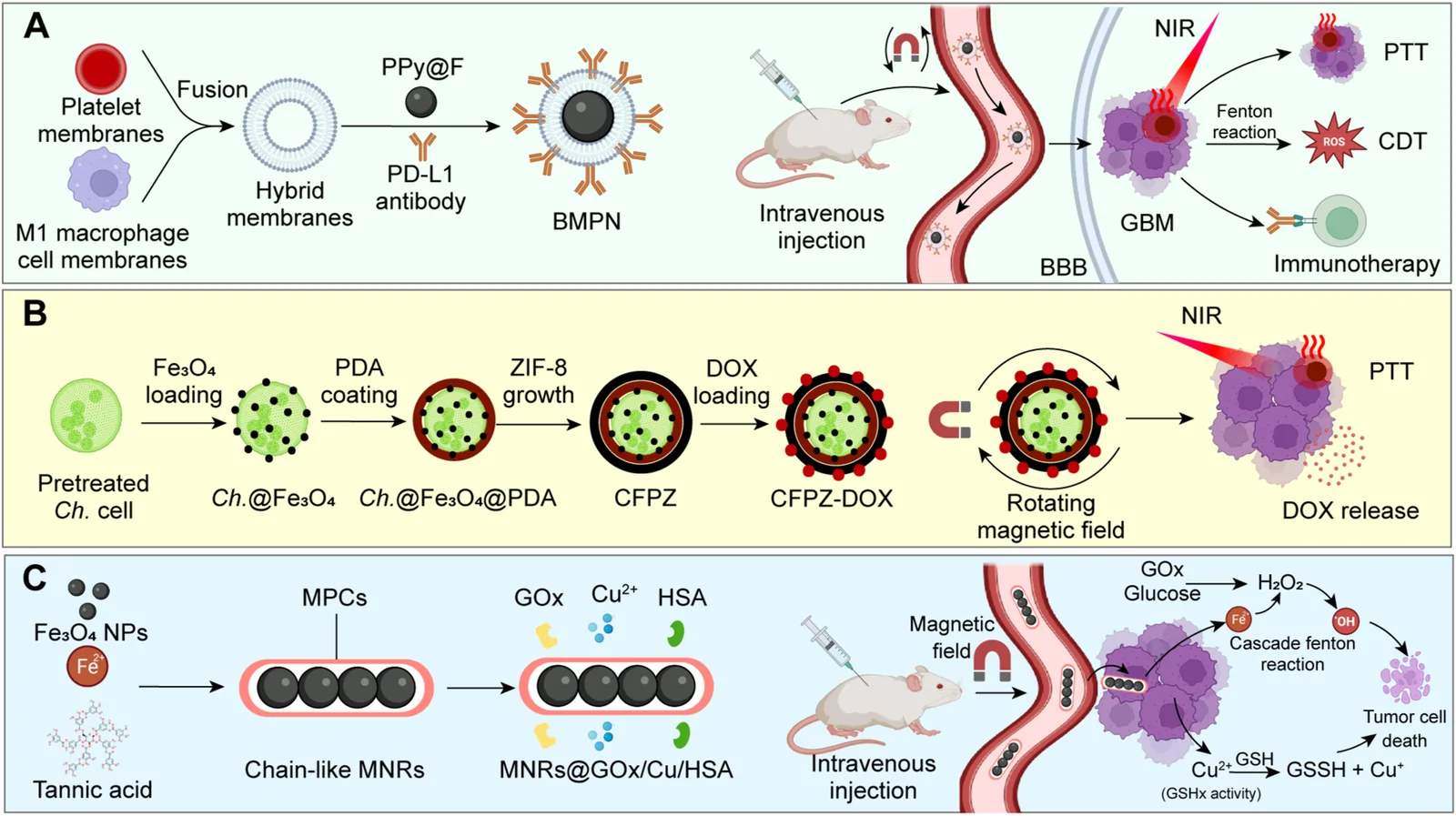
**MNRs with different treatment methods** (A) Hybrid membranes can be used as biomimetic capsules to encapsulate polypyrrole/Fe_3_O_4_ nanocomplex compounds (PPy@F), generating BMPNs. Using PD-L1/TMZ@BMPN NPs synergize photothermal/immunotherapy and promote Fenton reaction of Fe_3_O_4_. (B) Wrap PDA and ZIF-8 on the surface of *Ch.* magnetized with Fe_3_O_4_ to produce CFPZ. It can accurately advance in a rotating magnetic field and achieve rapid photothermal heating under the action of NIR. (C) Fe_3_O_4_ NPs, tannic acid, and Fe^2+^ were fixed by MPCs to form elongated chain-like MNRs. MPC coatings can be modified by GOD, Cu^2+^, and HSA, which can consume glucose and produce ·OH.

PTT is a highly promising treatment method, but it still faces challenges such as insufficient targeting efficiency and difficulty in precise temperature control. Excessive temperature can lead to adverse side effects and damage to normal cells, which may compromise therapeutic outcomes and, in some cases, potentially promote tumor progression or metastasis [167]. At present, research has proposed a clustered multifunctional heater-thermometer nanorobots (HT-NRs) capable of performing precise feedback heat transfer [157]. Cell experiments have confirmed that clusters of HT-NRs can actively penetrate microchannels and rapidly surround tumor cells in the target area. HT-NRs utilize their photothermal conversion and photothermal response color changes to regulate external NIR irradiation, achieving precise control of local hyperthermia and the affected area with high spatiotemporal resolution, ultimately achieving precise local PTT. This work may provide some inspiration for the development of intelligent multifunctional medical micro/nanorobots and their practical applications in precision disease treatment.

### 5.2 Chemodynamic therapy

As mentioned earlier, TME is very different from the normal human environment, mainly characterized by high H_2_O_2_, low oxygen and low pH [[168], [169], [170], [171]]. CDT is another emerging treatment method that relies on TME induced Fenton reaction. It refers to the use of transition metal ions (such as Fe^2+^ [172,173], Cu^2+^ [174] and Mn^2+^ [175,176]) to convert endogenous hydrogen peroxide in the TME into •OH, thereby causing oxidative stress in cancer cells [177,178]. This method converts the intrinsic substances in TME into toxic substances with significant therapeutic effects in situ, reducing toxicity to normal cells while killing tumor cells. It is gradually receiving more attention. Fe_3_O_4_ can serve as both a response to magnetic fields and induce Fenton reactions, and is widely used in the composition of many MNRs. For example, the Janus nanocatalytic robots (JNCRs) exhibited an increase in temperature and ROS production under NIR irradiation and iron-mediated Fenton reaction, leading to enhanced tumor treatment efficacy [179]. Experiments in mouse models have also demonstrated the safety of this nanorobot. Another study introduced metal polyphenol complexes (MPCs) into MNRs for tumor treatment. This type of MNRs is composed of Fe_3_O_4_ NPs, tannic acid, and Fe^2+^, resulting in a slender chain-like structure fixed by MPCs. These structures show a greater magnetic response than individual nanoparticles, offering flexibility in magnetic manipulation. The MPCs coating also allows for modification with glucose oxidase (GOx), Cu^2+^, human serum albumin (HSA), producing colloidally stable MNRs with a built-in multienzymatic cascade (MNRs@GOx/Cu/HSA) that consumes glucose, generates •OH, and depletes the antioxidant GSH (Fig. 4C) [180]. Cu^2+^ is also a commonly used transition metal ion for CDT, therefore, Cu^2+^ doped magnetic mesoporous silica NPs loaded with GOD (Cu@MPS-GOD) are built [181]. The MNRs can also activate immune cells or induce cancer cells to expose surface antigens, thereby promoting the immune system to kill cancer cells, producing systemic anti-tumor immunity [182].

In addition to utilizing transition metal ions, enzymes can also be linked to MNRs. For example, the JNCRs mentioned earlier can be connected to catalase, which can not only provide self-propelled power for MNRs by decomposing H_2_O_2_, but also improve the hypoxic environment of tumors [179]. Taking advantage of the fact that melanoma cells require a large extracellular supply of tyrosine, Chang et al. introduced a soluble Polyhemoglobin-Tyrosinase (PolyHb-Tyr) complex and nanoencapsulated it to form PolyHb-Tyr-Nano [183,184]. This drug inhibits tumor growth by depleting extracellular tyrosine. Further experiments have shown that PolyHb-Tyr-Nano, as an amino acid depletion therapy, can also inhibit other tumor cell lines. Based on this, Zhao et al. successfully constructed superparamagnetic Fe_3_O_4_ PolyHb-Tyr-Nano and demonstrated that by applying an external magnetic field and using a simple magnet, the superparamagnetic PolyHb-Tyr-Nano can be effectively retained after systemic clearance, significantly improving the effect of the drug on tumor cells. The effects of the drug include a reduction in amino acids, production of ROS, and a decrease in mitochondrial activity [185].

## 6 Current status of clinical translation of MNRs

### 6.1 Established precedents for the clinical translation of localized nanotherapies

As an emerging technology, it faces numerous challenges on the path toward clinical translation. Due to the lack of clinical trial data, answers to these questions cannot be obtained currently. Nevertheless, several nanotechnologies and physical therapeutic platforms that differ from MNRs in their action mechanisms have already advanced into clinical trials and accumulated experience that can be referenced in these aspects.

AuroLase® Therapy (NCT02680535) provides preliminary evidence for the clinical translation of MNRs. This trial used intravenous injection of gold silicon core-shell nanoparticles to verify the feasibility of the EPR effect in mediating passive tumor accumulation of nanoparticles in humans. The preliminary human data showed a lesion-level biopsy-negative rate of 87.5% at 12 months post-treatment, with no grade ≥3 adverse events reported [186]. Additionally, it established an operational paradigm for ablation guided by magnetic resonance imaging (MRI) and ultrasound fusion imaging. This trial provides direct evidence for the dual layer targeting strategy of MNRs, which relies primarily on magnetically controlled active targeting, supplemented by passive accumulation. Its imaging guidance template and the endpoint of biopsy negative rate are also transferable to the clinical evaluation system for MNRs. mRNA-4157 (V940) in combination with pembrolizumab (NCT03897881) utilizes lipid nanoparticles (LNPs) for the delivery of personalized neoantigens. In the KEYNOTE-942 phase IIb trial, the 2.5-year follow-up data showed that the recurrence-free survival rate increased from 55.6% in the monotherapy group to 74.8% in the combination group [187,188]. As a nano delivery platform validated by thousands of patients, systemic toxicity profile of LNP (liver accumulation, immunogenicity, metabolic half-life) provides a reliable baseline for predicting the safety of MNRs. The core distinction between MNRs and traditional nanomedicines lies in active physical regulation, and similar non-thermal ablation strategies have already received preliminary clinical validation on other physical therapeutic platforms. In the NCT04918381 Phase I trial, nanosecond pulsed-field ablation (nsPFA) technology (CellFX® system) was applied to treat superficial basal cell carcinoma (BCC). 92% of the BCC lesions showed complete histological clearance of BCC with no grade 3 or higher toxicity reported [189]. This trial demonstrated the effectiveness and safety of non-thermal physical ablation at the operational level, providing a feasible benchmark for the clinical translation of non-conventional physical regulation mechanisms. In addition to thermal ablation, the synergy between external field energy and nanomedicine can also be harnessed for radiosensitization. Nano-SMART (NCT04789486) is an ongoing adaptive Phase I/II trial designed to evaluate the safety and efficacy of AGuIX (gadolinium-based nanoparticles) combined with magnetic resonance (MR)-guided stereotactic body radiotherapy (SBRT) in centrally located lung tumors and locally advanced unresectable pancreatic ductal adenocarcinoma. The strategy of “nanodrug localization first, followed by external field irradiation” provides a reference framework for the clinical trial design of MNRs. The four trials described above all employed intravenous administration, whereas MAGNABLATE I (NCT02033447) explores another alternative administration strategy. This completed Phase 0 dose-escalation safety trial aims to evaluate the retention and distribution of magnetic iron oxide nanoparticles locally in the human body following intratumoral injection, thereby providing early human data for research on the in vivo behavior of magnetic nanomaterials. Real-time imaging tracking is indispensable for the clinical application of magnetic nanomaterials. The Ferumoxytol MRI trial (NCT00659126) has preliminarily demonstrated that Ferumoxytol (an SPION-based MRI contrast agent) offers the advantages of a prolonged imaging window and the absence of nephrotoxicity. This trial provides referential experience for MNRs to achieve theranostic integration. In summary, the aforementioned trials serve as reference examples for addressing several key issues related to MNRs and provide valuable experience for their clinical translation.

### 6.2 Preclinical progress in MNRs

However, the current in vivo experiments of MNRs are still at the stage of in vivo animal experiments. In addition to many small animal experiments, some experiments have been successfully verified in large animals. Landers et al. reported a magnetic control micro robot platform that can run under real clinical conditions [190], realizing the multi module integration from materials, navigation system, image visualization to targeted release, which is close to “clinical availability” in engineering degree for the first time. The platform is composed of a dual electromagnetic navigation system, a special release catheter and a biodegradable drug capsule robot, which can accurately control the microrobot in the clinical work area to move forward in the complex vascular network. In terms of the engineering system, the double Navion electromagnetic navigation system can generate magnetic field gradients up to 1 T/m at a constant navigation magnetic field of 30 mT. For acute thrombus treatment, the capsule completely dissolves at high temperature within 40 s under high-frequency magnetic stimulation (510 kHz at 20 mT) and releases the drug. Rolling on a liquid/solid interface achieved a peak forward velocity of 0.37 cm/s. Gradient-based pulling enabled motion against flow velocities up to 21.2 cm/s. In-flow navigation within a Y-junction achieved a successful capsule delivery rate of up to 95% for flows of 84 cm/s. The research team completed the whole process experiments of navigation, positioning and drug release in large animal models such as pigs and sheep. This achievement has laid a solid foundation for the future application in clinical scenarios such as stroke and refractory tumor.

The introduction of an external incentive source in tumor treatment to navigate the unbound drug-eluting micro robot in the blood can improve the selectivity of drug delivery. Li et al. Proposed an algorithm to predict the optimal patient position relative to gravity in the navigation process of intravascular microrobot [191]. In order to improve the targeting efficiency of magnetic microrobots, magnetic resonance navigation (MRN) using magnetic field gradient in clinical MRI was combined with the algorithm. In terms of engineering fabrication, the study employed superparamagnetic iron oxide nanoparticles with an average particle size of 12 ± 3.6 nm, a saturation magnetization of 70 emu/g, accounting for 60% of the total mass of the microrobots, resulting in a microrobot mass density of 2.95 g/cm^3^. To reduce blood flow resistance, a high-precision injector (with an injection accuracy of 0.00067 ml) and a balloon catheter system were used to precisely reduce the hepatic arterial flow rate from 3.3 ± 2.3 ml/s under free flow to 0.7 ± 0.3 ml/s (target range 0.5-1.0 ml/s). After optimization of body position and magnetic resonance navigation, the number of microrobots reaching the targeted lobe was 1.7 to 2.6 times that of the control group. Simulations based on 19 patients with hepatocellular carcinoma demonstrated that 78% (25/32) of the tumor nodules could be targeted by this method. This technology also offers a viable research avenue for the navigation of actuator-based human-scale microrobots.

Wu et al. developed a biomimetic reconfigurable nanorobotic complex (BRaNC) for precise intrathecal drug delivery in central nervous system (CNS) diseases [192]. The MNRs are composed of Fe_3_O_4_ NPs, model drugs (such as resolvin D2, RvD2), neutrophil membrane derived vesicles, and stem cell-derived exosomes, and possess magnetic responsiveness, a biomimetic interface, and drug delivery capabilities. Under the control of an external rotating magnetic field, the robot can self-organize into various group structures such as chain-like, vortex-like, and ribbon-like swarm in cerebrospinal fluid. In terms of engineering fabrication, this study constructed a BRaNC with an average particle size of 199.0 ± 3.3 nm. Through coating with neutrophil membrane vesicles and stem cell-derived exosomes, the macrophage uptake of iron was reduced from 17.8 ng per 1000 cells to 3.8 ng per 1000 cells. Under a 24 Hz alternating magnetic field, the vortex swarm achieved a maximum velocity of 44.7 μm/s, a minimum formation time of 6.0 s, and an aggregate size of approximately 0.2 mm. In terms of application outcomes, BRaNC-vortex treatment ultimately resulted in a 100% survival rate of spinal cord injury (SCI) mice over 15 days and restored their BMS score to 9.5 (close to normal). Meanwhile, microglial activation and proinflammatory cytokines were suppressed, while the levels of anti-inflammatory cytokines were elevated. This study is of great significance for the clinical translation of MNRs. The team not only validated the active targeting ability of the platform in a mouse model of spinal cord injury, but more importantly, built an intrathecal navigation platform that combines medical imaging and robotic arm-assisted magnetic field system in non-human primates. This achievement demonstrates the potential of the platform to overcome anatomical scale differences from rodents to primates, providing key experimental evidence for the translation of MNRs from animal models to human clinical practice.

MNR is the frontier direction of the cross integration of nanomedicine and intelligent materials. With the external magnetic field, it can control its movement and positioning in vivo noninvasively, in real time and accurately. It has shown great potential in tumor targeted drug delivery, magnetothermal therapy, diagnosis and treatment integration and other scenes. However, compared with the popularity of theoretical concepts and animal experiments, the clinical trials of “magnetic nanorobots” in the true sense are still in its infancy. In other words, to move from laboratory to clinic, MNRs still need to cross multiple thresholds, such as large-scale preparation, biosafety, in vivo behavior controllability and regulatory path.

## 7 Challenges and future perspectives in clinical translation

Although preclinical studies of MNRs have demonstrated impressive targeting efficiency and therapeutic indicators in small animal models, these achievements often rely on idealized conditions such as shallow tumor depth, uniform EPR-mediated vascular leakage, and genetically identical mouse strains [193]. These conditions cannot fully reflect human pathophysiology. In the following section, we will discuss the obstacles faced in clinical translation from the perspectives of biological barriers and immune safety, production, and imaging and visualization, and provide prospects for the future clinical translation of MNRs.

### 7.1 Biological barriers and immunological safety

Unlike traditional passive targeted nanomedicines, magnetic nanorobots can efficiently concentrate in the tumor vascular area through magnetic active navigation [194]. However, the subsequent extravasation from the vascular lumen across the endothelial barrier into the tumor stroma is still largely limited by tumor vascular permeability, and it is precisely at this stage that the huge gap in the EPR effect between preclinical models and human tumors constitutes a substantial bottleneck [195]. The accumulation of nanoparticles in tumors has sharply decreased in clinical studies compared with preclinical ones [196]. But the active targeting ability of MNRs can compensate for this gap to some extent. Magnetic driving can cause high concentrations of NPs to accumulate near the vascular wall, increasing their probability of contact and residence time on the endothelium, thereby partially offsetting the extravasation barrier caused by insufficient permeability [197]. Strategies such as penetrating targeted ligands, thermal and magnetic hyperthermia to enhance vascular permeability, and size-conversion designs responsive to the TME have been explored to address the remaining extravasation bottlenecks [[198], [199], [200]].

When discussing the immune safety of MNRs, protein corona is an unavoidable topic. Protein corona is a layer of protein “coat” that is instantly adsorbed on the surface of NMs/nanorobots after entering biological fluids, especially blood. The immune safety of MNRs is far more complex than that of single-component NPs. Its core contradiction is that the formation of protein coronas redefines the “biological identity” of MNRs, making their immunological properties no longer depends on the materials themselves, but depends on the opsonins (complement proteins, natural antibodies) and dysopsonins (albumin, apolipoproteins) in the protein corona [201]. The composition of the protein corona directly determines the recognition and clearance fate of macrophages towards magnetic nanorobots. The protein corona rich in opsonins is efficiently engulfed through Fc receptor/complement receptor mediation. The protein corona rich in dysopsonins confers immune escape ability [[202], [203], [204]]. The engulfed MNRs are not rapidly cleared by the body, but are slowly degraded by long-term retention in lysosomes of liver and spleen macrophages [205]. After being engulfed, MNRs degrade in lysosomes and release iron ions, which produce reactive oxygen species through Fenton reaction and affect macrophage polarization [206]. Moreover, the polarization state of macrophages themselves can also alter the composition of their surrounding protein corona, which in turn affects phagocytic efficiency [207]. Eventually, a dynamic cycle of mutual influence is formed. The phagocytosis of macrophages is not the endpoint of immune clearance, but the starting point of long-term liver and spleen accumulation and chronic toxicity of MNRs. The sustained phagocytosis of MNRs by reticuloendothelial system (RES) organs (liver, spleen) not only shortens their blood circulation time and weakens tumor targeting efficiency, but may also make these organs long-term reservoirs, posing a risk of chronic inflammation and immune cell dysfunction [208,209].

Currently, many materials with good biocompatibility have been applied in the construction of MNRs [113,[210], [211], [212], [213]]. However, in the face of these complex phenomena, the biocompatibility of a single component or simple system cannot be directly equated to the overall safety of multi-component composite MNRs. In fact, multi-component systems can cause interface synergistic toxicity that single component systems do not possess. Heterojunction effects, charge transfer, and surface chemical reconstruction between different materials may generate novel biological effects that cannot be predicted from individual safety data of each component [214,215]. Researchers have constructed a preliminary framework for addressing the multi-level safety challenges mentioned above from two dimensions: material design and evaluation methods. At the material design level, surface functionalization modification strategies such as PEGylation and cell membrane camouflage are used to prolong circulation time, reduce non-specific phagocytosis and protein adsorption [216,217]. The design of degradable materials (such as Enzymatically degradable gelatin methacryloyl (GelMA) and hydrolytically degradable poly (lactic-co-glycolic acid) (PLGA)) attempts to provide MNRs with a predictable degradation pathway in vivo to reduce the risk of long-term retention [218]. At the level of evaluation methods, the Safe by Design concept advocates incorporating safety considerations in the early stages of material design. The S.A.F.E. (Standardised characterisation, Assessment of biocompatibility, Facilitation of toxicity and exposure routes and Evaluation of clinical translation) framework provides systematic guidance for the evaluation of the entire process from characterization to clinical translation [219]. The stepwise screening scheme establishes a progressive validation pathway from in vitro to in vivo [220]. However, the above strategies are still in the conceptual validation stage, and the overall toxicity assessment criteria have not yet been established. There is currently no strategy to address the unpredictability of inter-individual immune differences, and the independent immunogenicity of MNRs degradation products has been almost unexplored [221,222]. In future clinical translation, it is necessary to establish a standardized evaluation system that covers long-term biological distribution, degradation kinetics, and chronic toxicity [223].

### 7.2 Engineering and manufacturing constraints in delivery systems

The engineering and large-scale production of MNRs face triple manufacturing bottlenecks, each of which profoundly restricts the transformation from laboratory prototypes to Good Manufacturing Practice (GMP)-level clinical supply. Clinical trials of SP1049C (a Pluronic micellar formulation loaded with DOX) also identified batch-to-batch variability, incomplete characterization across scales, and the absence of Process Analytical Technology (PAT) as major obstacles to the clinical approval of MNPs [224]. In terms of batch consistency, the physicochemical properties of MNPs are extremely sensitive to synthesis parameters such as temperature rise rate, precursor concentration, reagent addition kinetics, and purification sequence [225]. During the scale-up process, these parameters undergo fundamental changes in mixing mode, heat and mass transfer, and residence time distribution [226]. Consequently, the resulting particles exhibit significantly different particle size distribution, aggregation behavior, and magnetic characteristics compared to those expected from the same nominal formula [227,228]. For example, in microwave-assisted continuous flow synthesis, X-ray diffraction (XRD)-derived size and magnetic performance reproducibility are the best (90–95%), transmission electron microscopy (TEM) size similarity is 80–90%, while reaction yield reproducibility is less than 70% [229]. Two nominally identical commercial samples can exhibit significant differences in magnetic and thermal properties simply due to their different production dates [230]. Continuous flow/microfluidic systems can achieve reproducible synthesis with a core diameter of approximately 10 nm and a polydispersity index as low as 0.12, and Quality by Design (QbD) methods and multidimensional standardized analysis are being explored to control variability [231,232]. However, the loss of control in high-dimensional parameter space, the additional complexity of green synthesis, and the absence of PAT remain deep obstacles [[233], [234], [235]].

In terms of sterilization and preservation, the sterilization process itself can alter the stability, particle size, and zeta potential of NPs, and there is no universal method [236]. Gamma irradiation is feasible for specific MNPs systems, capable of preserving crystal structure, morphology, and size distribution, and fully suppressing microbial growth [237]. In contrast, ultraviolet (UV) sterilization combined with freeze-drying in the presence of PEG is most suitable for protein-coated particles [238]. However, the case dependence of method selection and the fragility of the multi-component structure of nanorobots make sterilization schemes highly complex [239].

In terms of GMP compliance, MNRs, as a “combination product” that combines drug, device, and biological product attributes, have a relatively vague regulatory positioning [240]. At present, the approval process for NP-based combination drugs (NBCDs) by the U.S. Food and Drug Administration (FDA) and the European Medicines Agency (EMA) has not been fully established [241]. Although the GMP batch production of SEONDex30 [242] has proven feasible and the approvals of Ferumoxytol and Magtrace® [243] provide a regulatory precedent. However, the delisting of Feridex due to safety concerns and better alternatives also warns that FDA approval itself does not guarantee long-term success [244]. The evaluation criteria for the unique properties of nanorobots (immune and electrophysiological effects of magnetic/electroactive components) still lack corresponding evaluation criteria in the current regulatory framework, which together constitute a systemic engineering bottleneck from the laboratory to the hospital bed [245].

### 7.3 Imaging and visualization

How to achieve real-time, high-resolution visualization and tracking in complex biological environments is an important challenge for the clinical translation of MNRs. At present, there is no single imaging modality that can meet all clinical requirements such as spatial resolution, temporal resolution, penetration depth, and safety [193]. Although MRI offers excellent soft tissue contrast and deep tissue imaging capabilities, the gradient amplitude of clinical gradient coils is limited, making it difficult to generate sufficient local magnetic force to drive MNRs. Additionally, its temporal resolution is insufficient for real-time tracking [246]. Ultrasound and photoacoustic imaging have good safety profiles [247], but distinguishing MNRs from background tissue signals remains difficult [248]. Fluorescence imaging, despite its high spatiotemporal resolution and molecular sensitivity, provides only shallow tissue penetration in the visible band and suffers from severe autofluorescence interference [249]. X-ray imaging can achieve real-time tracking, but it carries a risk of radiation exposure and its resolution is limited [250]. Laser speckle contrast imaging (LSCI) is constrained by optical scattering and absorption and is therefore only applicable to superficial vessels [251]. In addition, the magnetic material content must be balanced between magnetic responsiveness, imaging visibility, and cytotoxicity [252].

To overcome these challenges, researchers are vigorously developing multimodal imaging fusion strategies that integrate complementary modalities such as MRI, magnetic particle imaging (MPI), computed tomography (CT), and photoacoustic imaging, to achieve high-resolution anatomical localization and quantitative tracking [193]. As an emerging non-radioactive, high-sensitivity tomographic technique, MPI can quantitatively track the 3D spatial distribution of superparamagnetic NPs, offering a highly promising solution for real-time navigation of MNRs [[253], [254], [255]]. Given the inherent attenuation of magnetic fields in deep tissues, which compromises real-time steering authority, preoperative MRI-based 3D vascular reconstruction and intraoperative imaging feedback serve as essential compensatory strategies [190,256]. These enable trajectory planning and closed-loop control that partially offset penetration-induced actuation losses [257]. Deep learning and other artificial intelligence (AI) methods are also being used to improve the integration efficiency of multimodal imaging and the ability to recognize surgical boundaries [258]. However, the electromagnetic compatibility of imaging systems, light source delivery, system miniaturization, and seamless integration with magnetic drive systems remain practical challenges that constrain clinical implementation [259]. In the future, establishing a standardized imaging evaluation system that covers real-time navigation, long-term biological distribution monitoring, and degradation kinetics tracking will be a key direction for promoting the clinical translation of MNRs [193].

### 7.4 Outlook and future directions

The clinical translation of MNRs still needs to be promoted through multidimensional collaboration. Patient stratification should be given priority consideration [260]. Patient-derived xenograft models or organoid chips can be included in the prescreening workflow to quantify individual tumor vascular permeability, thereby identifying high-response patients and achieving biomarker-driven enrollment [261]. The scalability of manufacturing requires the adoption of a modular MNRs architecture, which consists of four independent and optimizable module: magnetic core, protective shell, stimulus-responsive unit, and targeting moiety [262]. This architecture has standardized interfaces, simplifying quality control from system validation to core parameter validation, and can adapt to continuous flow manufacturing platforms, minimizing variability between batches [263]. Long-term safety requires a half-life window of 2-6 weeks for degradation kinetics programming, with priority given to enzyme-triggered decomposition [264]. For example, tumor-overexpressed lipase can cleave poly(ε-caprolactone) (PCL) [265]. Active magnetic recycling can serve as a supplementary guarantee for non-degradable designs [266]. Deep tissue activation requires a multi-stage strategy. The gradient magnetic field to be used for overall targeting is combined with a rotating or traveling wave magnetic field for tissue penetration, supplemented by real-time imaging feedback (e.g., MRI), to form a closed-loop control system [267]. With the increasing maturity of preparation technology, continuous improvement of large-scale production capacity, and continuous promotion of multi-center clinical research, MNRs will open up a new technological route for precision tumor treatment, with extremely broad application prospects and huge commercial potential [193].

The deep integration of AI can help reshape MNRs from “external programmable devices” to “intelligent diagnostic and therapeutic entities” with autonomous perception, dynamic decision-making, and adaptive capabilities [255,268]. At the navigation level, model-free reinforcement learning enables microrobots to autonomously plan paths in complex fluid channels [269]. By combining deep learning algorithms for magnetic trajectory tracking with adaptive adjustment of AI planners, MNRs can achieve cloPATsed-loop intelligent control in dynamic in vivo environments [270]. Lin et al. demonstrated that their algal robot achieved a drug penetration enhancement of more than ten times in a mouse model of bladder cancer [271]. At the imaging monitoring level, deep learning algorithms can utilize two-dimensional MRI to achieve 3D spatial localization of MNRs, enabling intraoperative tracking to move from offline analysis to real-time closed-loop control [272]. At the material design level, the accuracy of hybrid deep learning models in predicting magnetic hyperthermia temperature has reached over 99.7% [273]. The physics-informed neural network has been applied for the first time in the prediction modeling of magnetic hyperthermia [274]. AI is combining patient-specific multi-omics data to transform tumor heterogeneity into quantitative design rules optimized for nanocarriers [275]. For drug release kinetics, Fathi-Karkan et al. employed Random Forest, Gradient Boosting, and Support Vector Regression models to interpolate pH-modulated oxaliplatin release profiles with R^2^ > 0.97, while SHAP analysis identified critical release phase transitions, demonstrating how machine learning can decipher complex cargo release mechanisms beyond simple curve-fitting [276]. The same research group further constructed a PIML framework that integrates physical laws—including DLVO theory and drug release kinetics—into data-driven models [277]. In terms of swarm intelligence, the introduction of AI enables the self-organizing behavior of magnetic micro-nano clusters in programmable fields to have autonomous coordination capabilities, and multiple MNRs can collaborate through communication to complete complex tasks [278]. In the future, the deep integration of AI and MNRs will continue to evolve along a closed-loop intelligent path of “perception-decision-execution” [268]. Challenges remain, including the robustness of algorithms in unpredictable biological environments, the computational demands for real-time data processing, and the urgent need to safeguard system data security [279]. Nevertheless, this fusion trend can enhance the ability of MNRs to perceive microenvironments in real time, dynamically plan paths, and autonomously adjust strategies, which is expected to bring new progress to the field of nanomedicine.

## 8 Conclusion

Here, we mainly discuss in detail the application of MNRs in tumor treatment and tumor treatment by carrying anticancer drugs. In PTT, MNRs have high efficiency of photothermal conversion. In terms of immunotherapy, it can enhance the anti-tumor effect by promoting the polarization of macrophages to the anti-tumor phenotype. In terms of drug delivery, with its nanometer size and magnetic driving characteristics, it can achieve targeted delivery and efficient release of drugs, reduce toxic and side effects and improve the therapeutic effect. Most of the components of MNRs are biocompatible materials. For example, they are often used to provide magnetic iron oxide components, which have low toxicity, can be degraded, and the metabolites are physiological iron. The materials used for the modification of MNRs are also easily degradable and biocompatible ingredients such as chitosan, silica and Cu^2+^. And the nano robot controlled by external magnetic field is also more friendly to the human body. This targeting method can actively target cancer tissues more accurately, release drugs with the help of controllable ways or special conditions of TME, improve the therapeutic effect and minimize the impact of drugs on healthy tissues. At present, a variety of drugs have shown their availability in animal experiments, but there is still pressure for clinical transformation. The effective and precise control of MNRs in vivo, the efficiency and targeting rate of drug delivery, and ultimately the therapeutic and prognostic effects of cancer need further exploration and research. With the gradual development and maturity of imaging technology and AI technology, combined with MNRs, we can more accurately locate the tumor site with the help of imaging, and improve and formulate personalized treatment plans with the help of AI technology. In a word, MNRs, as the cutting-edge achievements of nanotechnology, medicine, material science and other interdisciplinary intersection, are showing great application potential and broad development prospects.

## CRediT authorship contribution statement

**Xiaoyu Liu:** Writing – review & editing, Writing – original draft, Visualization, Validation, Investigation, Data curation, Conceptualization. **Changying Li:** Writing – review & editing, Supervision, Software, Methodology, Funding acquisition. **Zhijie Xu:** Writing – review & editing, Writing – original draft, Validation, Supervision, Software, Resources, Investigation, Formal analysis, Data curation, Conceptualization. **Yuanliang Yan:** Writing – review & editing, Validation, Resources, Project administration, Formal analysis, Data curation, Conceptualization. **Wangrui Liu:** Writing – review & editing, Visualization, Validation, Supervision, Resources, Data curation, Conceptualization. **Yong Li:** Writing – review & editing, Visualization, Project administration, Methodology, Investigation, Data curation, Conceptualization. **Ming Gao:** Writing – review & editing, Supervision, Resources, Project administration, Funding acquisition, Formal analysis, Conceptualization.

## Funding information

This study was supported by the 10.13039/501100001809National Natural Science Foundation of China (Grant No. U24A20771, 22222611, 82403784), the Scientific Research Cultivation Project in Emerging Strategic Fields (202404), the Youth Innovation Team Program of Colleges and Universities in Shandong Province (2025KJJ013), the Talent Introduction Project of Shandong First Medical University (045RC-200019).

## Declaration of competing interest

The authors declare that they have no known competing financial interests or personal relationships that could have appeared to influence the work reported in this paper.

## Data Availability

No data was used for the research described in the article.
